# Differentiation of multiple system atrophy from Parkinson’s disease by structural connectivity derived from probabilistic tractography

**DOI:** 10.1038/s41598-019-52829-8

**Published:** 2019-11-11

**Authors:** Alexandra Abos, Hugo C. Baggio, Barbara Segura, Anna Campabadal, Carme Uribe, Darly Milena Giraldo, Alexandra Perez-Soriano, Esteban Muñoz, Yaroslau Compta, Carme Junque, Maria Jose Marti

**Affiliations:** 1 Medical Psychology Unit, Department of Medicine, Institute of Neuroscience, University of Barcelona, Barcelona, Catalonia Spain; 2 Centro de Investigación Biomédica en Red sobre Enfermedades Neurodegenerativas (CIBERNED), Hospital Clínic de Barcelona, Barcelona, Catalonia Spain; 3 Movement Disorders Unit, Neurology Service, Hospital Clínic de Barcelona. Institute of Neuroscience,University of Barcelona, Barcelona, Catalonia Spain; 4 Institute of Biomedical Research August Pi i Sunyer (IDIBAPS), Barcelona, Catalonia Spain

**Keywords:** Machine learning, Parkinson's disease

## Abstract

Recent studies combining diffusion tensor-derived metrics and machine learning have shown promising results in the discrimination of multiple system atrophy (MSA) and Parkinson’s disease (PD) patients. This approach has not been tested using more complex methodologies such as probabilistic tractography. The aim of this work is assessing whether the strength of structural connectivity between subcortical structures, measured as the number of streamlines (NOS) derived from tractography, can be used to classify MSA and PD patients at the single-patient level. The classification performance of subcortical FA and MD was also evaluated to compare the discriminant ability between diffusion tensor-derived metrics and NOS. Using diffusion-weighted images acquired in a 3 T MRI scanner and probabilistic tractography, we reconstructed the white matter tracts between 18 subcortical structures from a sample of 54 healthy controls, 31 MSA patients and 65 PD patients. NOS between subcortical structures were compared between groups and entered as features into a machine learning algorithm. Reduced NOS in MSA compared with controls and PD were found in connections between the putamen, pallidum, ventral diencephalon, thalamus, and cerebellum, in both right and left hemispheres. The classification procedure achieved an overall accuracy of 78%, with 71% of the MSA subjects and 86% of the PD patients correctly classified. NOS features outperformed the discrimination performance obtained with FA and MD. Our findings suggest that structural connectivity derived from tractography has the potential to correctly distinguish between MSA and PD patients. Furthermore, NOS measures obtained from tractography might be more useful than diffusion tensor-derived metrics for the detection of MSA.

## Introduction

Multiple system atrophy (MSA) is a sporadic, progressive neurodegenerative disease characterized by autonomic failure, parkinsonian symptoms, and/or cerebellar features. Striatonigral degeneration and olivopontocerebellar atrophy are typically found at postmortem examination of MSA patients, reflecting the presence of parkinsonian features and ataxia, respectively^1^. Although MSA may resemble Parkinson’s disease (PD) in its early stages, brain damage is more aggressive, with usually no response to dopaminergic medication, and leading to a rapidly progressive disease course with a fatal prognosis^1,2^. For this reason, improving our ability to diagnose and to predict MSA progression after diagnosis is a major objective in clinical practice.

Conventional diagnostic magnetic resonance imaging (MRI) has been widely used as a complementary tool in the differential diagnosis between PD and MSA. In most cases of PD, clinical MR imaging shows no abnormalities until advanced disease stages, and brain degeneration is typically not as extensive as in MSA^3^. Typical radiological features in MSA are mainly located in subcortical structures, including a cruciform hyperintensity in the pons, called the “hot cross bun sign”; changes in the putamen comprising atrophy and T2 signal hypointensity, with a marginal hyperintensity; and atrophy of the cerebellar peduncles (chiefly the middle cerebellar peduncle (MCP)), pons, and cerebellum^4^.

The use of 3 T MR scanning alongside advanced analysis techniques has led to improvements in the diagnostic value of MRI. Diffusion-weighted MRI (DWI), especially using diffusion tensor imaging (DTI), is one of the most common MRI techniques when studying neurodegenerative diseases, as it allows detecting microstructural abnormalities and assessing the integrity of white matter (WM) tracts. Previous DTI studies in MSA have characterized diffusion abnormalities with different approaches such as using regions of interest (ROI) and tract-based spatial statistics (TBSS). Disrupted WM integrity, decreased streamline density and abnormal diffusivity measures were observed in subcortical structures such as the basal ganglia, middle cerebellar peduncles, cerebellum, and pons^5–8^.

Tractography allows reconstructing brain WM pathways, which helps understand how the brain operates as a connected system. Furthermore, apart from quantifying the local streamline density, tractography can be used to reconstruct the structural connectome – i.e., a comprehensive description of the structural connections between brain regions^9^. In recent years, structural connectivity has been studied in PD patients, showing reduced structural connectivity between the substantia nigra and the striatum and thalamus in these patients^10–12^. Furthermore, reduced fiber density has been observed between the associative and limbic cortex, putamen, thalamus, caudate, and globus pallidus in PD compared with controls^13^. Given that PD is a very heterogeneous disease with both motor and non-motor symptoms, structural connectivity has also been used to study subgroups with different predominant symptomatology. Structural connectivity differences were observed in PD with and without tremor^14^, freezing of gate^15–17^, PD-MCI^18^, and different motor subtypes^19^. However, although studying the connectome has proven useful to detect structural abnormalities in PD, as far as we know, limited work has been done in terms of characterizing MSA connectivity pattern using tractography^20,21^.

With the introduction of machine learning algorithms, MRI studies have been able to test the importance of specific measures in discriminating different diseases or conditions. As DTI has been useful in characterizing subcortical abnormalities in MSA, diffusion measures such as fractional anisotropy (FA) and mean diffusivity (MD) have been used as features to differentiate between PD and MSA patients. Sensitivities and specificities around 80% have been achieved in most studies^22–25^. These results seem to indicate that diffusion tensor-derived metrics might be useful for discriminating between MSA and PD. FA and MD are commonly used to detect microstructural abnormalities in subcortical structures, but no information about the relationship between regions can be obtained from these measures. Tractography allows assessing whether the connectivity between these structures is also impaired, which is relevant to understand the pathological pathways of neurodegenerative diseases. Thus, tractography-derived metrics might be of interest to identify specific abnormal brain connections with higher discriminating power. To the best of our knowledge, no previous published works focused on combining structural connectivity and machine learning to discriminate PD from MSA patients.

In the present study, we use tractography to discriminate patients with MSA from patients with PD. Our hypothesis is that structural connectivity between subcortical structures is informative enough to distinguish MSA from PD at the individual-subject level. To test this hypothesis, we passed the connectivity data into a supervised machine learning algorithm and assessed its ability to correctly determine each patient’s group membership. Additionally, we hypothesize that subcortical structural connectivity derived from tractography is more informative than previously studied diffusion tensor-derived metrics.

## Results

### Subjects

Table 1 shows the sociodemographic and clinical characteristics of the samples. No significant intergroup differences were observed for age and years of education (p = 0.3317 and p = 0.3803, respectively). A significant intergroup effect was observed for gender distribution (p = 0.0436).

**Table 1:** Sociodemographic and clinical characteristics by group.

	HC (n = 54)	PD (n = 65)	MSA (n = 31)	Stat/p
Age	64.3 (11.3)	65.4 (10)	60.9 (8.4)	2.0434/p = 0.3317
Years of education	12.63 (4.3)	12.48 (5.4)	10.68 (3.8)	1.9333/p = 0.3803
Sex (male/female)	26/28	48/17	19/12	4.2921/p = 0.0436*^1^
Years of evolution	—	8.26 (6.02)	4.46 (2.75)	3.3466/p < 0.001*^3^
H&Y (1:2:3:4:5)	—	10:32:22:1:0	0:8:11:9:3	5.77/p < 0.001*^3^
UPDRS	—	16.59 (9.22)	—	—
UMSARS	—	—	49.97 (20.46)	—
LEDD	—	639.35 (382.76)	520.67 (426.4)	1.347/p = 0.0936

HC: healthy controls; PD: Parkinson’s disease patient group; MSA: multiple system atrophy patient group; H&Y: Hoehn and Yahr scale; UPDRS: United Parkinson’s Disease Rating Scale, motor section scores; UMSARS: United Multiple System Atrophy Rating Scale, motor section scores; LEDD: levodopa equivalent daily dose (in mg); * refers to significant results; Post-hoc differences between HC and PD^1^; HC and MSA^2^; PD and MSA^3^.

Comparing the two groups of patients, no significant differences were found between PD and MSA patients for age, years of education, gender, or levodopa equivalent daily dose (LEDD) (p = 0.0668, p = 0.1234, p = 0.3141, and p = 0.0936 respectively). MSA patients differed from PD participants only in disease duration and Hoehn and Yahr scores (H&Y) (both p < 0.001).

Although patients showed no significant demographical differences, these characteristics could influence and bias the performance of the classifier. We therefore repeated the classification algorithm using a better matched subsample of PD and MSA patients with equal sample sizes (n = 30 in both groups) and no significant differences in age, years of education, gender, or LEDD (p = 0.1969, p = 0.7203, p = 0.8223, and p = 0.1692 respectively) (Supplementary Table 1). Concretely, PD patient were randomly sampled based on the proportion of male/female patients and the demographic characteristics of MSA patients.

### Structural connectivity analysis

**Figure 1:**
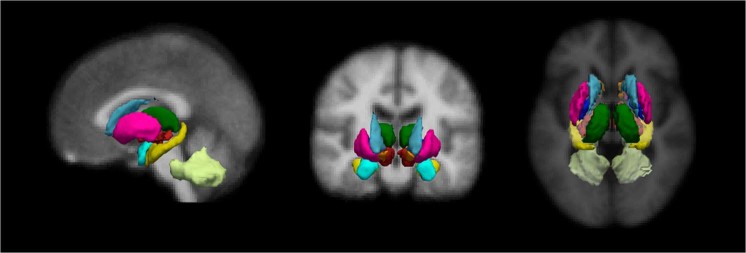
Eighteen subcortical region of interest (ROI) from FreeSurfer. Representation of the subcortical parcellation used in this study (for right and left hemispheres): the bilateral nucleus accumbens; amygdala; caudate nucleus; hippocampus; pallidum; putamen; thalamus; ventral diencephalon (including the hypothalamus, mammillary body, subthalamic nuclei, substantia nigra, red nucleus) and cerebellar white matter, including the middle cerebellar peduncles.

**Figure 2:**
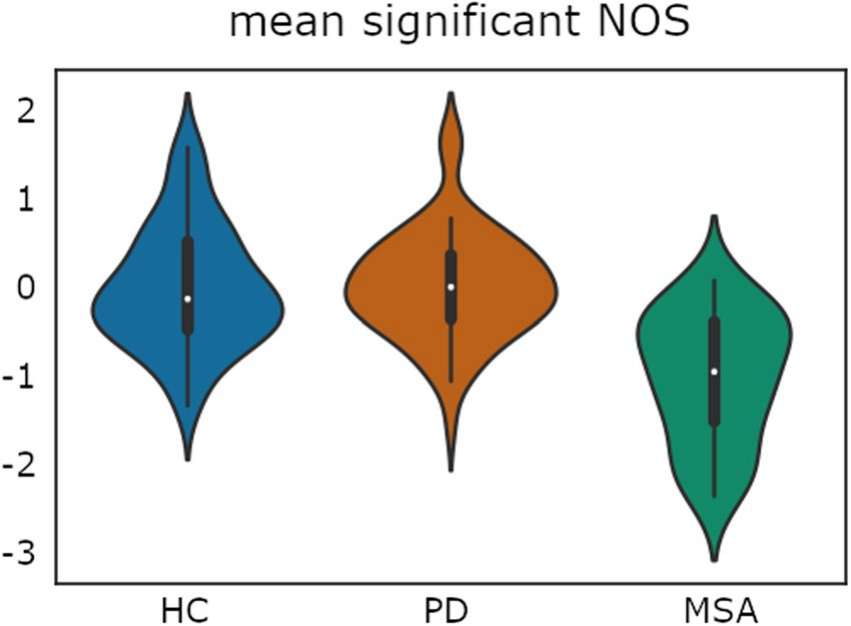
Plot illustrates the distribution of the average number of streamlines (NOS) between the 10 significantly reduced tracts found in MSA patients using threshold-free network based statistics (TFNBS). NOS values were Z-transformed to calculate the global mean value comprising all significant connections; HC: healthy controls; PD: Parkinson’s disease group; MSA: multiple system atrophy group. Plot width represents the frequency (density) of values; the height indicates the upper (max) and lower (min) limits.

**Figure 3:**
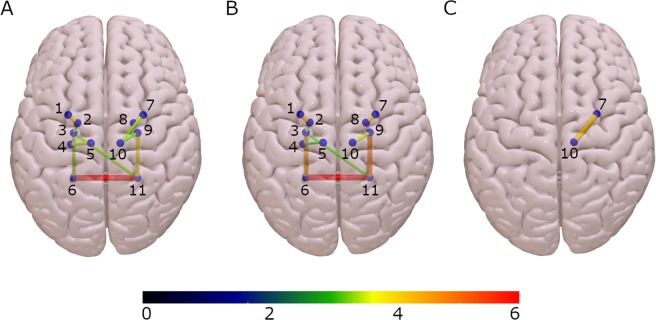
Connectivity differences between groups patients using threshold-free network based statistics (TFNBS). (**A**) Connectivity differences between healthy controls and multiple system atrophy patients. (**B**) Connectivity differences between Parkinson’s disease and multiple system atrophy patients. (**C**) Connectivity differences between healthy controls and Parkinson’s disease patients. Brain edges are scaled according to the value of the T statistic (shown in the color bar), p < 0.05, FDR corrected. 1: Left Putamen; 2: Left Pallidum; 3: Left Ventral diencephalon; 4: Left Hippocampus; 5: Left Thalamus; 6: Left Cerebellum; 7: Right Putamen; 8: Right Pallidum; 9: Right Ventral diencephalon; 10: Right Thalamus; 11: Right Cerebellum.

The nodes used to construct the structural connectivity network were derived from the automated FreeSurfer segmentation^26,27^, which includes 18 subcortical structures (Fig. 1). The connectivity between regions was represented as the number of streamlines between them (NOS). Significant differences in NOS were found between groups in 10 subcortical connections (Fig. 2 and Table 2) using a method called threshold-free network based statistics (TFNBS)^28^, which performs statistical inference on brain graphs. TFNBS works similarly to network-based statistics (NBS)^29^ but without requiring the definition of a component-defining threshold and generating edge-wise significance values. The subcortical structures linked to connections showing reduced NOS in the MSA group compared with healthy controls (HC) and PD were the putamen, the pallidum, the ventral diencephalon, the thalamus, and the cerebellum, in both right and left hemispheres (Supplementary Fig. 1). Post-hoc testing showed reduced connectivity in all 10 connections in MSA patients compared with HC (Fig. 3A) and in eight connections in MSA compared with PD patients (Fig. 3B). No connections showed significantly higher NOS in PD or MSA patients compared with HC, or in MSA compared with PD subjects. In PD patients compared with HC, only the connection between the putamen and the thalamus in the right hemisphere was found to be significantly reduced (Fig. 3C).

**Table 2:** Significant reduced connections found with TFNBS by group.

	HC (n = 54)	PD (n = 65)	MSA (n = 31)	Stat/p
Left Pallidum-Left Putamen	1.4874·10^6^ (0.2522·10^6^)	1.5197·10^6^ (0.2627·10^6^)	1.2184·10^6^ (0.2599·10^6^)	F = 5.1788/p < 0.001^2,3^*
Left Hippocampus-Left Thalamus	1.023·10^6^ (0.2549·10^6^)	0.9295·10^6^ (0.2975·10^6^)	0.7847·10^6^ (0.2147·10^6^)	F = 2.4171/p = 0.0092^2,3^*
Left Pallidum-Left VentralDC	1.1713·10^6^ (0.4372·10^6^)	1.2162·10^6^ (0.4139·10^6^)	0.8819·10^6^ (0.3154·10^6^)	F = 3.6096/p = 0.0065^2,3^*
Left VentralDC-Left Cerebellum	0.7598·10^6^ (0.2172·10^6^)	0.8399·10^6^ (0.2092·10^6^)	0.5747·10^6^ (0.2734·10^6^)	F = 5.2403/p < 0.001^2,3^*
Right Pallidum-Right Putamen	1.3556·10^6^ (0.2265·10^6^)	1.4148·10^6^ (0.2195·10^6^)	1.1536·10^6^ (0.3044·10^6^)	F = 4.7647/p < 0.001^2,3^*
Right Putamen-Right Thalamus	0.7936·10^6^ (0.2888·10^6^)	0.6769·10^6^ (0.2435·10^6^)	0.5442·10^6^ (0.3885·10^6^)	F = 1.9587/p = 0.0065^1,2^*
Right Thalamus -Right VentralDC	2.7867·10^6^ (0.4611·10^6^)	2.8886·10^6^ (0.4822·10^6^	2.4535·10^6^ (0.5392·10^6^)	F = 3.8987/p = 0.0042^2,3^*
Left VentralDC-Right Cerebellum	0.0918·10^6^ (0.0623·10^6^)	0.0834·10^6^ (0.0503·10^6^)	0.0471·10^6^ (0.0426·10^6^)	F = 3.1419/p = 0.0096^2,3^*
Left Cerebellum -Right Cerebellum	4.4335·10^6^ (1.4225·10^6^)	4.1623·10^6^ (1.2313·10^6^)	2.3522·10^6^ (1.032·10^6^)	F = 6.7853/p < 0.001^2,3^*
Right VentralDC-Right Cerebellum	0.8462·10^6^ (0.2694·10^6^)	0.9045·10^6^ (0.2629·10^6^)	0.5694·10^6^ (0.2649·10^6^)	F = 5.722/p < 0.001^2,3^*

HC: healthy controls; PD: Parkinson’s disease patient group; MSA: multiple system atrophy patient group; * significant FDR corrected results; Post-hoc differences between HC and PD^1^; HC and MSA^2^; PD and MSA^3^; Stats refers to F-test (F).

### ROI analysis

Diffusion tensor-derived metrics (FA and MD) extracted from the 18 subcortical ROIs were evaluated as a measure of microstructural integrity. Significant FA differences surviving FDR correction were found in the bilateral ventral diencephalon, left amygdala, right thalamus, and right cerebellum. Significant MD differences were detected in the nucleus accumbens, putamen and ventral diencephalon in the left hemisphere, and in the pallidum, thalamus and cerebellum in the right hemisphere. Post-hoc testing showed diffusion abnormalities (reduced FA and increased MD) in all structures in the MSA group compared with the HC and PD samples, except for the left nucleus accumbens, where no MD increments were found between MSA and PD. Abnormal FA and MD values in PD compared with HC were found in the thalamus and the putamen. Furthermore, increased FA was detected in MSA patients compared with HC and PD patients in the left amygdala. A summary of the significant FA and MD results is shown in Tables 3 and 4. A detailed description is given in Supplementary Tables 2 and 3.

**Table 3:** Significant Fractional Anisotropy (FA) of subcortical ROIs by group.

	HC (n = 54)	PD (n = 65)	MSA (n = 31)	Stat/p
Left Amygdala	0.2585 (0.019)	0.2535 (0.020)	0.2689 (0.029)	F = 5.6173/p = 0.0144*^2,3^
Left Ventral DC	0.4327 (0.024)	0.4279 (0.029)	0.4116 (0.027)	F = 7.1122/p = 0.004*^2,3^
Right Thalamus	0.4218 (0.024)	0.4155 (0.025)	0.3997 (0.026)	F = 9.7117/p = 0.0018*^1–3^
Right Ventral DC	0.4731 (0.033)	0.4766 (0.037)	0.4462 (0.030)	F = 9.1112/p = 0.0024*^2,3^
Right Cerebellum	0.3351 (0.026)	0.3357 (0.020)	0.2884 (0.041)	F = 37.0711/p = 0.0018*^2,3^

HC: healthy controls; PD: Parkinson’s disease patient group; MSA: multiple system atrophy patient group; * significant FDR corrected results; Post-hoc differences between HC and PD^1^; HC and MSA^2^; PD and MSA^3^; Stats refers to F-test (F).

**Table 4:** Significant Mean Diffusivity (MD) of subcortical ROIs by group.

	HC (n = 54)	PD (n = 65)	MSA (n = 31)	Stat/p
Left Accumbens	8.755·10^−4^ (5.5·10^−5^)	9.095·10^−4^ (6.7·10^−5^)	9.213·10^−4^ (7.7·10^−5^)	F = 4.5752/p = 0.0354*^1,2^
Left Putamen	7.739·10^−4^ (4.5·10^−5^)	7.914·10^−4^ (4.9·10^−5^)	8.161·10^−4^ (7.8·10^−5^)	F = 6.9336/p = 0.009*^1–3^
Left Ventral DC	1.206·10^−3^ (9.9·10^−5^)	1.232·10^−3^ (1.1·10^−4^)	1.313·10^−3^ (1·10^−4^)	F = 10.3436/p = 0.0012*^2,3^
Right Pallidum	7.725·10^−4^ (4.3·10^−5^)	7.794·10^−4^ (4.5·10^−5^)	8.084·10^−4^ (7.9·10^−5^)	F = 5.2247/p = 0.0212*^2,3^
Right Thalamus	1.24810^−3^ (1·10^−4^)	1.271·10^−3^ (1.1·10^−4^)	1.350·10^−3^ (1.1·10^−4^)	F = 9.2948/p = 0.0012*^2,3^
Right Cerebellum	9.187·10^−4^ (7.5·10^−5^)	9.162·10^−4^ (7.1·10^−5^)	1.141·10^−3^ (1.9·10^−4^)	F = 55.287/p = 0.0012*^2,3^

HC: healthy controls; PD: Parkinson’s disease patient group; MSA: multiple system atrophy patient group; * significant FDR corrected results; Post-hoc differences between HC and PD^1^; HC and MSA^2^; PD and MSA^3^; Stats refers to F-test (F).

Intergroup subcortical volume comparisons were also performed to explore the presence of atrophy in these structures (Supplementary Table 4). All regions except for the left thalamus showed significant effects, surviving FDR correction. The volume of these structures was significantly reduced in MSA patients compared with HC and PD patients, except for the amygdala, where no difference was found between MSA and PD patients. Significant volumetric reductions were observed in PD compared with HC in the bilateral nucleus accumbens and the left amygdala. No significant volumetric reductions were detected in HC compared with either patient group.

### Classification

As a leave-one-out cross-validation (LOOCV) scheme was used to avoid overfitting by not using the test subject in the selection of features, we calculated a set of significant features at each iteration to pass them into the classification algorithm. Intergroup comparison followed by recurrent feature selection were employed to select the informative features to be introduced into the classifier. All 10 subcortical connections that showed significant group effects when using the whole sample were obtained in more than 80% of the iterations. Therefore, these connections seem to be stable in the feature selection procedure. The SVM classification algorithm correctly predicted overall group membership with an accuracy of 0.78, with a sensitivity of 0.71, and a specificity of 0.86.

Considering the imbalanced group sizes and the differences in gender distribution in the overall sample, we repeated the entire procedure (feature selection and classification) using a matched subsample of PD patients and MSA patients, achieving an overall accuracy, sensitivity, and specificity of 0.84, 0.77, and 0.90, respectively.

Additionally, we also evaluated the classification performance between MSA patients and HC. The SVM classifier achieved an overall accuracy, sensitivity, and specificity of 0.79, 0.84, and 0.74, respectively.

Given that previous studies assessed the classification ability of other features extracted from subcortical structures such as diffusion tensor-derived metrics (e.g., FA and MD) and structure volume, we evaluated the performance of these metrics as well. A summary of these results is shown in Table 5. For these metrics, the previously described feature selection and classification procedure was employed. Subcortical FA and MD measures performed worse than NOS, concretely displaying a lower sensitivity. On the other hand, volumetric information from subcortical ROIs achieved a higher classification performance in all comparisons. Additionally, we evaluated whether combining multimodal features, in this case NOS and volumetric information, would improve the discrimination of MSA patients. We observed that introducing both sets of measures improved the sensitivity when differentiating MSA from HC.

**Table 5:** Discrimination performance of diffusion and structural features by groups.

	NOS	DT-metrics	SC vol	NOS + SC-vol
**MSA vs PD**				
Accuracy	0.79	0.75	0.84	0.84
Sensitivity	0.71	0.58	0.74	0.74
Specificity	0.86	0.92	0.94	0.94
**MSA vs HC**				
Accuracy	0.79	0.72	0.85	0.89
Sensitivity	0.84	0.61	0.77	0.84
Specificity	0.74	0.82	0.93	0.94
**MSA vs PD subsample**				
Accuracy	0.84	0.76	0.90	0.87
Sensitivity	0.77	0.68	0.84	0.81
Specificity	0.90	0.84	0.97	0.94

HC: healthy controls; PD: Parkinson’s disease patient group; MSA: multiple system atrophy patient group; NOS: number of streamlines, DT-metrics: diffusion tensor-derived metrics, SC vol: subcortical volume.

## Discussion

In this work, using a probabilistic connectivity approach, we have found evidence of reduced structural connectivity between subcortical structures in MSA patients compared with HC and with PD patients. We have also achieved a high accuracy in discriminating between PD and MSA patients using subcortical edge-wise tractography data and supervised machine learning.

We observed significant connectivity alterations in MSA patients, in agreement with previous findings and with our a priori hypothesis. Decreased NOS in MSA patients compared with both PD patients and HC was found in connections linked to basal ganglia such as the pallidum and putamen, the ventral diencephalon, the thalamus, and the cerebellum white matter/MCP.

As complementary analyses, we assessed diffusion tensor-derived metrics (FA and MD) and volumes of the 18 subcortical ROIs. In agreement with our structural connectivity results and previous studies^30–34^, we found abnormal diffusion tensor-derived measures and atrophy in the putamen, the pallidum, the ventral diencephalon, the thalamus, and the cerebellum. Concretely, we identified the cerebellum as the most damaged structure in terms of volumetric atrophy and abnormal diffusion measures, which agrees with the connectivity results as the connection between left and right cerebellum shows the most significant reduction of number of streamlines (NOS) between MSA patients and the other study groups. The vulnerability of the cerebellum in this disease is well-known and has been extensively described in previous studies^30,35–37^. Olivopontocerebellar atrophy is a hallmark of MSA, resulting in the characteristic ataxia. Abnormal basal ganglia volume, mostly in the putamen, is another common MSA feature^36,38–41^, as striatonigral degeneration is also detected at postmortem examination in MSA patients. Thus, our findings of reduced NOS and atrophy involving the cerebellum and striationigral structures are in agreement with the previously described neuropathology of MSA.

While conventional DTI approaches, such as voxel-wise comparisons of FA and MD maps, have been previously used in characterizing MSA and distinguishing it from other diseases, limited work has been done in terms of assessing structural connectivity of MSA patients derived from tractography^20,21,42,43^. Thus, as our findings cannot be directly compared with previous published work, more studies should be performed to assess the generalizability of these results.

Regarding the PD group, no connectivity reductions were observed when compared with MSA patients, in agreement with previous studies^6,30,44–46^. Compared with HC, on the other hand, PD patients showed weakened connections between the putamen and the thalamus in the right hemisphere, as also previously described^10,47–49^. Connectivity results in PD agree with diffusion tensor-derived measures, as we found reduced FA in the putamen and thalamus in PD patients compared with HC, which is an indicator of microstructural disruption. Although previous studies have also reported subcortical atrophy of putamen and nucleus accumbens in PD^36,50,51^, in our sample, we were only able to detect volume reduction in the nucleus accumbens. Moreover, the strongest NOS reduction in MSA compared with PD was also the inter-cerebellar connection. Although cerebellar abnormalities have been reported in PD^52^, the cerebellum is not a key structure in the degenerative process of this disease. In this study, PD participants exhibit no detectable abnormalities in this structure. Concretely, PD patients did not differ from HC in inter-cerebellar connections, diffusion-tensor derived metrics nor cerebellar volume.

Considering these findings, it is difficult to speculate whether gray matter damage is the primary feature, subsequently leading to loss of white matter structural connectivity between subcortical regions, or whether the degenerative process of the MSA disease directly involves fiber abnormalities. Complementarily, the 10 connections found significantly reduced in MSA were correlated with subcortical volumes, to assess whether connectivity results were derived by the volumetric reduction. Only the NOS between the putamen and pallidum in the left hemisphere correlated with subcortical volumes (r = 0.34, p = 0.031). Therefore, although ROIs size might have an effect in NOS, the connectivity results do not seem to be derived exclusively by this factor.

Given that MSA patients can be incorrectly diagnosed as having PD, especially in early stages of the disease, the development of neuroimaging biomarkers that can distinguish between these two illnesses with a high accuracy at the individual patient level would be of great clinical interest. Based on the vulnerability of certain subcortical structures since early phases of the MSA degenerative process, we hypothesized that the number of streamlines between these regions might have the ability to distinguish MSA from PD patients with a high accuracy. We found that the streamline count in the affected connections detected using edge-wise connectivity analysis was informative enough to distinguish MSA patients from PD patients with satisfactory accuracy, concretely of 0.78 and 0.84, when using the whole dataset and a paired subsample, respectively. The selection of the brain connections used in this classification procedure was done through a combination of edge-wise intergroup comparisons and a recurrent feature elimination method. To avoid circularity, this step was nested in the LOOCV procedure, thereby never including the test subject in the comparison that defined the features used to train the classifier. Additionally, discrimination between MSA and HC shown accurate performance as well, concretely an overall accuracy of 0.79.

Given that NOS measures showed a satisfactory classification performance, we qualitatively evaluated whether these features derived from tractography outperformed the diffusion tensor-derived metrics employed in previous studies^24,25,32^. For this reason, we complementarily assessed the discriminant ability of subcortical FA and MD measures. In our sample, diffusion tensor-derived metrics showed worse discrimination performance compared with the NOS features. These results suggest that tractography might provide more discriminant information than FA and MD. Additionally, we also assessed the classification performance of subcortical volumes, as previous studies also described their potential discriminant ability. The overall results showed that volume features outperformed the discrimination obtained through NOS analysis. However, it is noteworthy that the combination of volumetric and NOS features resulted in a better discrimination between MSA and HC. These results are in agreement with previous work highlighting the classification improvement observed when combining multimodal MRI data^25^.

Taken together, our results demonstrate the usefulness of NOS for correctly distinguishing MSA from PD and HC, which outperformed the classification obtained with diffusion tensor-derived metrics. Although volumetric information was shown to be slightly superior to tractography measures, the combination of both sets of features improved the discrimination ability of the classifier.

The discriminating power of the connectivity features described in this study must also be demonstrated in future studies using samples of early-stage patients. Since abnormalities in subcortical structures occur early in MSA, this approach may prove to be appropriate in this setting. However, it is important to highlight that, although having shown promising results, for the moment, tractography methods are still not ready to be integrated in clinical routine. These techniques are very time-consuming and require visual inspection to ensure the quality of some steps, as well as technical knowledge to interpret the desired measurements. Therefore, more user-friendly platforms are required to integrate this methodology as part of a clinical diagnostic procedure.

Some limitations to the present study should be further addressed. First, given the relatively small sample size of MSA patient, we cannot assume that the set of discriminating network connections found in this study would be identified in other datasets; consequently, a larger sample is required to further validate these results. Complementary, a larger dataset would have allowed the use of k-fold cross-validation, a technique with smaller variance compared to the current leave-one-out cross-validation^53^.

Thirdly, in this study we performed a qualitative evaluation of multimodal models; thus, we cannot conclude whether the combination of different MRI features is an optimal approach to distinguish MSA from PD patients. In this context, further work should be carried out to find the most optimal combination of features and to quantitatively study the performance of this specific combination of multimodal data.

Fourthly, using subcortical gray matter structures as seeds is still challenging for diffusion tractography, as the tissue has an influence in the construction of the network, i.e.- using WM ROIs as seeds generates stronger networks with a larger number of connections than seeding in gray matter ROIs^54^. Finally, the characteristics of the reconstructed tracts are widely affected by the tractography techniques and parcellations employed. However, reduced integrity of subcortical connections in MSA patients has been extensively reported in previous MSA studies, which supports the validity of our findings.

## Conclusion

In this work, we found evidence that WM structural connectivity between the cerebellum and the basal ganglia is weakened in MSA patients compared with controls and PD patients. Our results also suggest that these measures of connectivity may allow discriminating between MSA and PD patients at the individual patient level with a high accuracy, indicating a potential usefulness of this approach as a tool for differential diagnosis.

## Methods

### Participants

Thirty-eight MSA patients and sixty-seven PD patients were recruited from the Parkinson’s Disease and Movement Disorders Unit, *Hospital Clínic de Barcelona*. Fifty-six healthy controls were recruited from *the Institut de l’Envelliment, Universitat Autònoma d*e Barcelona and from friends or patients’ spouses who volunteered to participate in the study. Detailed information of the sample can be found in our previous work^55^.

The inclusion criteria for patients were: (i) the fulfillment of the UK PD Society Brain Bank diagnostic criteria for PD and (ii) the fulfillment of the Gilman criteria^56^ for MSA.

Exclusion criteria consisted of: (i) MRI movement artifacts, (ii) pathological MRI findings other than mild WM hyperintensities or suggestive of alternative diagnoses in patients, (iii) significant neurological, systemic, or psychiatric comorbidity in the HC and PD groups, (iv) Mini-Mental State Examination scores < 25 or dementia according to Movement Disorder Society criteria.

Two PD patients, six MSA patients, and one HC were excluded for excessive movement. One MSA patient was excluded for MR artifacts. One HC was excluded for WM hyperintensities. The final sample therefore consisted of 54 HC, 31 MSA patients, and 65 PD patients.

Motor disease severity was evaluated using the Unified Multiple System Atrophy Rating Scale (motor section) for MSA patients, and the Unified Parkinson’s Disease Rating Scale (motor section) for PD patients.

Written informed consent was obtained from the participants after full explanation of the procedures involved. The study was approved by the Ethics Committee of the Hospital Clinic and the University of Barcelona (HCB/2015/0798 and IRB00003099, respectively) and it was performed in accordance with relevant regulations and guidelines.

### Basic statistical analyses

As described in our previous work^57^, intergroup comparisons of demographic, clinical, and imaging variables were performed with the general linear model using in-house MATLAB scripts. Statistical significance was established through the Monte Carlo simulations with 10000 permutations. Two-tailed p-values were calculated as the proportion of values in the null distribution more extreme than those observed in the actual model. FDR was then used to control for multiple comparisons. In all analyses, gender was included as a covariate of no interest. Spearman correlations between clinical and imaging variables were evaluated using SPSS-24 (2016; Armonk, NY: IBM Corp.).

### MRI acquisition

MRI data were acquired with a 3 T Siemens scanner (MAGNETOM Trio). The scanning protocol included high-resolution 3-dimensional T1-weighted images acquired in the sagittal plane (TR = 2300 ms, TE = 2.98 ms, TI = 900 ms, 240 slices, FOV = 256 mm; 1 mm isotropic voxel), an axial FLAIR sequence (TR = 9000 ms, TE = 96 ms), and two sets of single band spin-echo diffusion-weighted MRI in the axial plane with opposite phase-encoding directions (anterior-posterior (AP) and posterior-anterior (PA)) (TR = 7700 ms, TE = 89 ms, FOV = 244 mm; 2 mm isotropic voxel). Diffusion-weighted images were acquired along 30 directions with a b value = 1000 s/mm^2^. An image without diffusion weighting (b = 0 s/mm^2^) was also acquired. Both sets of images were acquired at both AP and PA phase-encoding directions.

### MRI preprocessing

Structural MRI preprocessing was performed using the automated FreeSurfer pipeline (version 5.1; https://surfer.nmr.mgh.harvard.edu/), which includes several independent steps: removal of non-brain tissue, automated Talairach transformation, intensity normalization^58^, tessellation of the gray matter/WM boundary, automated topology correction^59^, and surface deformation to optimally place the gray matter/WM and gray matter/cerebrospinal fluid boundaries^27^. The output of each step was visually inspected to guarantee correct and accurate preprocessing.

Manual inspection was initially performed to identify motion-related and intensity artifacts in the DWI images, which were subsequently preprocessed with FSL (version 5.08; https://fsl.fmrib.ox.ac.uk/fsl) using the FDT (FMRIB’s Diffusion Toolbox)^60^. The DWI pipeline included several steps: brain extraction, susceptibility-induced distortion correction, and eddy-current distortion and subject motion correction. After the preprocessing, a diffusion tensor model was then fit at each voxel to the 4D DWI data series using DTIFit^61^. Fractional anisotropy (FA) and mean diffusivity (MD) maps were obtained as outputs of this process.

### Regions of interest

Eighteen subcortical structures/regions (Fig. 1) were selected as regions of interest (ROIs) using the automated FreeSurfer segmentation^26,27^: bilateral nucleus accumbens; amygdala; hippocampus; caudate nucleus; putamen; pallidum; thalamus; ventral diencephalon (including the hypothalamus, mammillary body, subthalamic nuclei, substantia nigra, red nucleus, lateral geniculate nucleus, and medial geniculate nucleus); and cerebellar white matter, including the middle cerebellar peduncles (MCP). These ROIs, generated in native structural space, were linearly registered to native diffusion space with FSL’s Flirt in order to be used as seeds. The FA map from each subject was used as a reference image to compute the affine transformation from structural space to diffusion space using correlation ratio as a cost function. This transformation was then applied to the subcortical ROIs to transform them to native diffusion space. The resulting images were visually checked to assess the quality of the transformation. Each voxel was uniquely assigned to the mask with highest probability of membership to ensure that ROI masks did not overlap with each other due to resample blur.

### DTI and volumetry data of subcortical structures (ROIs)

Measures of mean FA and MD were calculated for each subcortical ROI to evaluate the microstructural diffusion properties of the 18 subcortical structures. Complementarily, the FreeSurfer volume-based stream^62^ was used to extract the volume (in mm^3^) of each ROI, which is often used as a marker of atrophy. Gender was included as a covariate in all analyses. Intracranial volume was also used as a covariate in the volumetric comparisons.

### Brain network computation

The probability distribution of fiber directions in each voxel was calculated with Bedpostx^63^, which models diffusion signal as ball and stick components to generate a distribution of likely fiber orientations within each voxel^61^. Bedpostx runs Markov Chain Monte Carlo sampling to build up distributions on diffusion parameters at each voxel that can then be repeatedly sampled to allow voxel-to-voxel propagation of streamlines until stopping criteria are met^63^. Probtrackx is used to compute the probabilistic connectivity between seeds^63^. It repeatedly samples from the principal diffusion direction probability distribution calculated in Bedpostx, at the voxel-wise level, creating a new streamline at each iteration^64^. For each seed region, 5000 streamlines were grown from each voxel with tracking parameters of 0.5 mm step size, 2000 maximum number of steps and a ±80° curvature threshold. The strength of structural connectivity between each pair of regions was obtained from the number of reconstructed streamlines (NOS) between these ROIs. This process was repeated for all ROI pairs, to compute an 18 × 18 subcortical connectivity matrix. For each pair of ROIs, the total number of streamlines were considered: those starting from i to j, and those initiated from j to i, thus obtaining a symmetric structural connectivity matrix. As described in our previous work^57^, to reduce the risk of false-positive connections, streamlines intersecting fewer than two regions were ignored, and only connections between pairs of regions that were detected in at least 50% of subjects were considered^65^.

### Characterization of structural connectivity differences

In order to test for intergroup differences in interregional NOS between PD, MSA, and HC, we used TFNBS^28^. This approach combines threshold-free cluster enhancement, a method frequently used in voxel-wise statistical inference^66^, and network-based statistics^29^, commonly used for statistical analysis of brain graphs. Initially, the symmetric 18 × 18 connectivity matrices M of all individual subjects underwent group testing, where an F statistic was calculated for each entry *i*, *j* using the general linear model, producing a group 18 × 18 raw F statistic matrix Mstat. With TFNBS, the raw statistic value of each Mstat edge is replaced by its TFNBS score. Concretely, this score is determined by the heights of its neighboring edges (extension) and by the strength of the statistical effect (height); thus, the final TFNBS scores are influenced by how topologically “clustered” these effects are. Finally, a p value is ascribed to each entry in the TFNBS-enhanced matrix through permutation testing. Further description of this method can be found in Baggio *et al*. (2018).

Gender was included in the intergroup connectivity analyses as a covariate of no interest, and control of the false discovery rate (FDR) to 5% was used to correct for multiple testing across the connectome. Post-hoc comparisons were then tested in the significant connections found with TFNBS. Connectivity Fig. 3 was drawn using Surf Ice (www.nitrc.org).

### Classification algorithm

LOOCV was implemented to avoid circularity in the classification (PD vs. MSA). When applying LOOCV, one subject is used for testing whereas the algorithm training is performed with the remaining N-1 subjects. This cross-validation technique enhances the generalization power of the classifier and prevents overfitting^67^.

**Figure 4:**
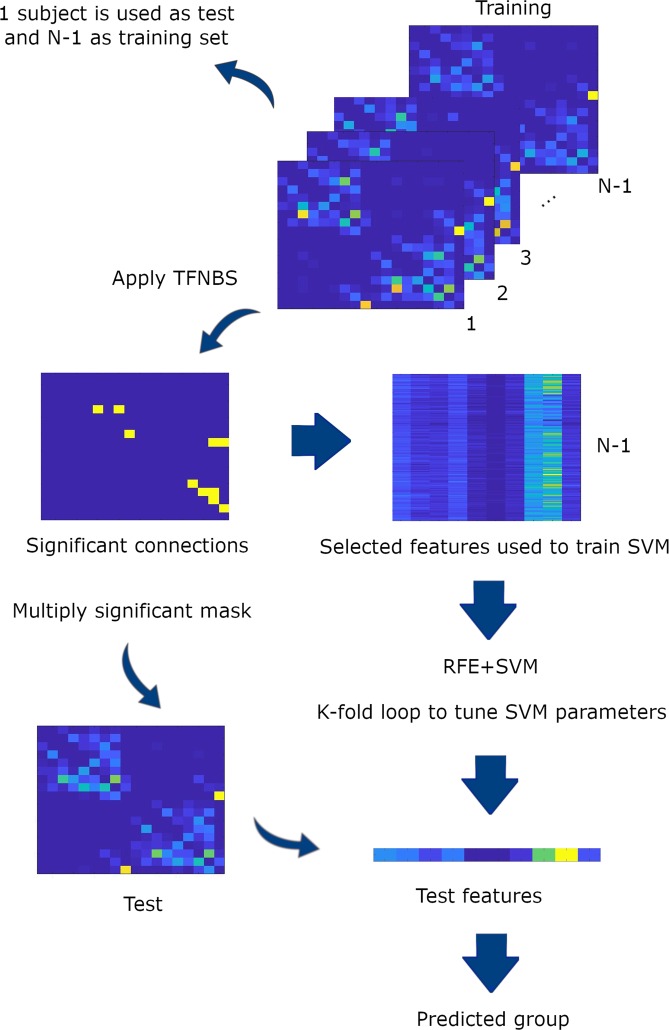
Classification procedure. Representation of one iteration of the feature selection and machine learning procedure. For each iteration, one subject was defined as the test set, whereas the remaining N-1 subjects made up the training set. Each training set was then fed into the TFNBS to calculate the significant connections between groups. Subsequently, the significant connections were introduced into a recursive feature elimination (RFE) algorithm to select the optimal connections. The support vector machine (SVM) algorithm was then tuned with the selected features. The resulting classifier model was then used to classify the corresponding test subject.

In order to select the most important features, a mixed feature selection method was used for dimensionality reduction. Firstly, an intergroup comparison with TFNBS was computed to select an initial set of features. Secondly, a recursive feature elimination (RFE) method was employed to find the most optimal features to be introduced into the classifier (see Fig. 4). Finally, these features were entered into the classification algorithm to train the classifier. To test whether they were informative enough to differentiate PD from MSA patients, the trained algorithm was then applied to the test subject, with the whole process repeated N times. A detailed description of this method can be found in Abos *et al*. (2019).

The classification was assessed with a linear support vector machine (SVM), concretely using the SVM function from scikit-learn (http://scikit-learn.org), implemented in Python. Additionally, a C parameter needed to be defined, which determines the tradeoff between a correct classification of training examples and the maximization of the decision function’s margin. In order to choose the best C parameter in an unbiased fashion, a cross-validated grid-search was employed.

The performance of the classification procedure was evaluated with the following measures: (i) accuracy (number of subjects correctly classified as PD or MSA patients divided by total number of subjects), (ii) sensitivity (number of MSA patients correctly classified divided by the total number of MSA patients), and (iii) specificity (number of PD patients correctly classified divided by the total number of PD patients). When assessing the ability of the classifier in discriminating between MSA patients and HC, the specificity referred to the number of HC correctly classified divided by the total number of HC.

## Supplementary information

Supplementary Document

## Data Availability

The data that support the findings of this study are available from the corresponding author upon reasonable request.
