# Supplementary material for: Differentiation of multiple system atrophy from Parkinson’s disease by structural connectivity derived from probabilistic tractography
Source: Sci Rep. 2019 Nov 11;9:16488. doi: 10.1038/s41598-019-52829-8 (PMC6848175; doi:10.1038/s41598-019-52829-8)
Supplement: Supplementary file 1 — Supplementary Document [file 41598_2019_52829_MOESM1_ESM.pdf]

Supplementary data

Differentiation of multiple system atrophy from Parkinson's disease by structural connectivity derived from probabilistic tractography

Alexandra Abos[1], Hugo C. Baggio[1], Barbara Segura[1], Anna Campabadal[1], Carme Uribe[1], Darly M Giraldo[2,3], Alexandra Perez-Soriano[2,3], Esteban Muñoz[2,3], Yaroslau Compta[2,3], Carme Junque[1,2,4], Maria Jose Marti[2,3]

[1]Medical Psychology Unit, Department of Medicine. Institute of Neuroscience, University of Barcelona.Barcelona, Catalonia, Spain.

[2]Centro de Investigación Biomédica en Red sobre Enfermedades Neurodegenerativas (CIBERNED),Hospital Clínic de Barcelona. Barcelona, Catalonia, Spain.

[3]Movement Disorders Unit, Neurology Service, Hospital Clínic de Barcelona. Institute of Neuroscience,University of Barcelona, Barcelona, Catalonia, Spain.

[4] Institute of Biomedical Research August Pi i Sunyer (IDIBAPS). Barcelona, Catalonia, Spain.

***Corresponding author:** Dr. Maria Jose Marti

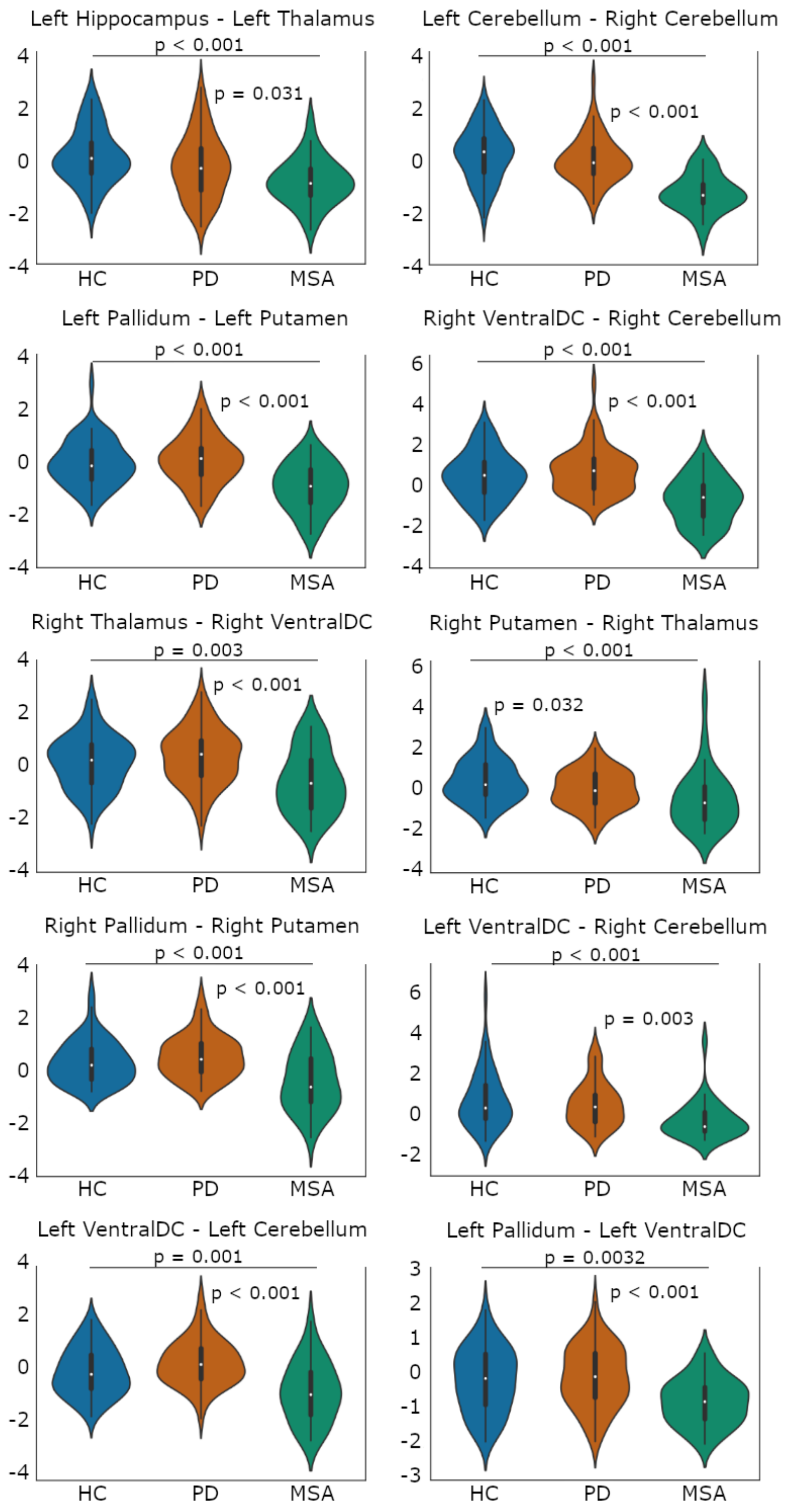

Supplementary figure 1. Plot illustrates the distribution of the number of streamlines (NOS) between the 10 significantly reduced tracts found in MSA patients using threshold-free network based statistics (TFNBS). NOS values were Z-transformed; HC: healthy controls; PD: Parkinson's disease group; MSA: multiple system atrophy group.

Supplementary Table 1. Sociodemographic and clinical parameters by group of the balanced sample of PD and MSA patients used for the classification procedure.

	PD (n=31)	MSA (n=31)	Stat/ p
**Age**	64.55 (8.3)	60.9 (8.4)	1.70/p=.1969
**Years of education**	11.58 (5.4)	10.68 (3.8)	0.76/p=.7203
**Sex (male/female)**	21/10	19/12	0.52/p=.8223
**Years of evolution**	10.39 (6.55)	4.46 (2.75)	4.64/p<.001*
**H&Y (1:2:3:4:5)**	5:15:11:0:0	0:8:11:9:3	4.84/p<.001*
**LEDD**	629.07 (414.1)	520.67 (426.4)	0.9902/p=.1692

PD: Parkinson's disease patient group; MSA: multiple system atrophy patient group; H&Y: Hoehn and Yahr scale; LEDD: levodopa equivalent daily dose (in mg); * refers to significant results; Post-hoc differences between HC and PD[1]; HC and MSA[2]; PD and MSA[3];

Supplementary Table 2. Fractional Anisotropy (FA) of subcortical ROIs by group.

	HC (n=54)	PD (n=65)	MSA (n=31)	Stat/ p
**Left Accumbens**	0.2737 (0.020)	0.2705 (0.023)	0.2756 (0.021)	F=1.5978/ p=0.2618
**Left Amygdala**	0.2585 (0.019)	0.2535 (0.020)	0.2689 (0.029)	F=5.6173/ p=0.0144*[2,3]
**Left Caudate**	0.2865 (0.023)	0.2929 (0.027)	0.2987 (0.034)	F=2.0163/ p=0.2529
**Left Hippocampus**	0.2596 (0.021)	0.2525 (0.023)	0.2512 (0.022)	F=1.8303/ p=0.2598
**Left Pallidum**	0.4283 (0.042)	0.4303 (0.042)	0.4359 (0.038)	F=0.4489/ p=0.6749
**Left Putamen**	0.3771(0.027)	0.3728 (0.023)	0.3796 (0.024)	F=1.7513/ p=0.2598
**Left Thalamus**	0.3768 (0.020)	0.3816 (0.022)	0.3775 (0.022)	F=0.3641/ p=0.6929
**Left Ventral DC**	0.4327 (0.024)	0.4279 (0.029)	0.4116 (0.027)	F=7.1122/ p=0.004*[2,3]
**Left Cerebellum**	0.2894 (0.027)	0.2878 (0.021)	0.2896 (0.033)	F=0.5908/ p=0.6311
**Right Accumbens**	0.2490 (0.021)	0.2421 (0.018)	0.2523 (0.024)	F=3.8997/ p=0.0537
**Right Amygdala**	0.2733 (0.023)	0.2753 (0.029)	0.2843 (0.040)	F=1.4372/ p=0.2896
**Right Caudate**	0.2593 (0.022)	0.2503 (0.022)	0.2560 (0.028)	F=1.9789/ p=0.2529
**Right Hippocampus**	0.4100 (0.041)	0.4053 (0.037)	0.4291(0.047)	F=4.1309/ p=0.0516
**Right Pallidum**	0.3710 (0.021)	0.3679 (0.021)	0.3757 (0.028)	F=1.9794/ p=0.2529
**Right Putamen**	0.3756 (0.018)	0.3776 (0.021)	0.3696 (0.023)	F=1.6627/ p=0.2618
**Right Thalamus**	0.4218 (0.024)	0.4155 (0.025)	0.3997 (0.026)	F=9.7117/ p=0.0018*[1,2,3]
**Right Ventral DC**	0.4731 (0.033)	0.4766 (0.037)	0.4462 (0.030)	F=9.1112/ p=0.0024*[2,3]
**Right Cerebellum**	0.3351 (0.026)	0.3357 (0.020)	0.2884 (0.041)	F=37.0711/ p=0.0018*[2,3]

HC: healthy controls; PD: Parkinson's disease patient group; MSA: multiple system atrophy patient group; * refers to FDR corrected results; Post-hoc differences between HC and PD[1]; HC and MSA[2]; PD and MSA[3]; Stats refers to F-test (F).

Supplementary Table 3. Mean Diffusivity (MD) of subcortical ROIs by group.

	HC (n=54)	PD (n=65)	MSA (n=31)	Stat/ p
**Left Accumbens**	$8.755\cdot10^{-4}$ ($5.5\cdot10^{-5}$)	$9.095\cdot10^{-4}$ ($6.7\cdot10^{-5}$)	$9.213\cdot10^{-4}$ ($7.7\cdot10^{-5}$)	F=4.5752/ p=0.0354*[1,2]
**Left Amygdala**	$9.838\cdot10^{-4}$ ($8.3\cdot10^{-5}$)	$9.890\cdot10^{-4}$ ($9.3\cdot10^{-5}$)	$9.521\cdot10^{-4}$ ($5.9\cdot10^{-5}$)	F=2.1035/ p=0.2153
**Left Caudate**	$1.262\cdot10^{-3}$ ($1.2\cdot10^{-4}$)	$1.265\cdot10^{-3}$ ($1\cdot10^{-4}$)	$1.256\cdot10^{-3}$ ($9\cdot10^{-5}$)	F=0.2476/ p=0.8758
**Left Hippocampus**	$1.144\cdot10^{-3}$ ($9.8\cdot10^{-5}$)	$1.192\cdot10^{-3}$ ($1.1\cdot10^{-4}$)	$1.181\cdot10^{-3}$ ($9\cdot10^{-5}$)	F=2.179/ p=0.2153
**Left Pallidum**	$7.674\cdot10^{-4}$ ($6.8\cdot10^{-5}$)	$7.688\cdot10^{-4}$ ($6.8\cdot10^{-5}$)	$7.664\cdot10^{-4}$ ($7.2\cdot10^{-5}$)	F=0.4114/ p=0.8082
**Left Putamen**	$7.739\cdot10^{-4}$ ($4.5\cdot10^{-5}$)	$7.914\cdot10^{-4}$ ($4.9\cdot10^{-5}$)	$8.161\cdot10^{-4}$ ($7.8\cdot10^{-5}$)	F=6.9336/ p=0.009*[1,2,3]
**Left Thalamus**	$1.057\cdot10^{-3}$ ($5.9\cdot10^{-5}$)	$1.061\cdot10^{-3}$ ($6.4\cdot10^{-5}$)	$1.062\cdot10^{-3}$ ($6.4\cdot10^{-5}$)	F=0.0322/ p=0.9711
**Left Ventral DC**	$1.206\cdot10^{-3}$ ($9.9\cdot10^{-5}$)	$1.232\cdot10^{-3}$ ($1.1\cdot10^{-4}$)	$1.313\cdot10^{-3}$ ($1\cdot10^{-4}$)	F=10.3436/ p=0.0012*[2,3]
**Left Cerebellum**	$8.4\cdot10^{-4}$ ($4.8\cdot10^{-5}$)	$8.647\cdot10^{-4}$ ($5.2\cdot10^{-5}$)	$8.531\cdot10^{-4}$ ($4.8\cdot10^{-5}$)	F=3.2145/ p=0.1098
**Right Accumbens**	$9.816\cdot10^{-4}$ ($6.8\cdot10^{-5}$)	$9.964\cdot10^{-4}$ ($7.7\cdot10^{-5}$)	$9.924\cdot10^{-4}$ ($8.1\cdot10^{-5}$)	F=0.6145/ p=0.7417
**Right Amygdala**	$1.274\cdot10^{-3}$ ($1.2\cdot10^{-4}$)	$1.288\cdot10^{-3}$ ($1.1\cdot10^{-4}$)	$1.292\cdot10^{-3}$ ($9\cdot10^{-5}$)	F=0.162/ p=0.8974
**Right Caudate**	$1.154\cdot10^{-3}$ ($9.6\cdot10^{-5}$)	$1.2\cdot10^{-3}$ ($1.1\cdot10^{-4}$)	$1.192\cdot10^{-3}$ ($9.9\cdot10^{-5}$)	F=2.0399/ p=0.2232
**Right Hippocampus**	$7.888\cdot10^{-4}$ ($7.8\cdot10^{-5}$)	$7.878\cdot10^{-4}$ ($6.8\cdot10^{-5}$)	$7.786\cdot10^{-4}$ ($9\cdot10^{-5}$)	F=0.4367/ p=0.8082
**Right Pallidum**	$7.725\cdot10^{-4}$ ($4.3\cdot10^{-5}$)	$7.794\cdot10^{-4}$ ($4.5\cdot10^{-5}$)	$8.084\cdot10^{-4}$ ($7.9\cdot10^{-5}$)	F=5.2247/ p=0.0212*[2,3]
**Right Putamen**	$1.077\cdot10^{-3}$ ($6.8\cdot10^{-5}$)	$1.084\cdot10^{-3}$ ($6.3\cdot10^{-5}$)	$1.101\cdot10^{-3}$ ($5.4\cdot10^{-5}$)	F=1.4494/ p=0.3609
**Right Thalamus**	1.24810^{-3} ($1\cdot10^{-4}$)	$1.271\cdot10^{-3}$ ($1.1\cdot10^{-4}$)	$1.350\cdot10^{-3}$ ($1.1\cdot10^{-4}$)	F=9.2948/ p=0.0012*[2,3]
**Right Ventral DC**	$9.019\cdot10^{-4}$ ($1\cdot10^{-4}$)	$9.047\cdot10^{-4}$ ($1.1\cdot10^{-4}$)	$9.6\cdot10^{-4}$ ($1.1\cdot10^{-4}$)	F=3.0339/ p=0.1222
**Right Cerebellum**	$9.187\cdot10^{-4}$ ($7.5\cdot10^{-5}$)	$9.162\cdot10^{-4}$ ($7.1\cdot10^{-5}$)	$1.141\cdot10^{-3}$ ($1.9\cdot10^{-4}$)	F=55.287/ p=0.0012*[2,3]

HC: healthy controls; PD: Parkinson's disease patient group; MSA: multiple system atrophy patient group; * refers to FDR corrected results; Post-hoc differences between HC and PD[1]; HC and MSA[2]; PD and MSA[3]; Stats refers to F-test (F).

Supplementary Table 4. Volumetric measures (mm^3) of subcortical ROIs by group.

	HC (n=54)	PD (n=65)	MSA (n=31)	Stat/ p
**Left Accumbens**	525.80 (102.4)	495.08 (110.5)	446.84 (128)	F=6.313/p=.0038*[1,2]
**Left Amygdala**	1588.57 (227.66)	1540.45 (304.28)	1464.26 (225)	F=4.751/p=.0171*[1,2]
**Left Caudate**	3345.61 (419.37)	3403.82 (528.38)	3065.97 (569.47)	F=4.894/p=.0102*[2,3]
**Left Hippocampus**	4055.56 (450.62)	3992.66 (627.99)	3692.87 (443.2)	F=6.018/p=.0038*[2,3]
**Left Pallidum**	1653.59 (228.69)	1771.55 (278.02)	1374.03 (285.88)	F=24.226/p<.001*[2,3]
**Left Putamen**	5048.76 (565.34)	5013.09 (767.11)	4140.52 (1121.98)	F=17.326/p<.001*[2,3]
**Left Thalamus**	6360.06 (640.82)	6643.49 (837.85)	6218.10 (710.86)	F=2.96/p=.0542
**Left Ventral DC**	3939.56 (448.72)	4179.68 (531)	3520.13 (500.58)	F=19.789/p<.001*[2,3]
**Left Cerebellum**	16474.33 (3715.37)	16862.20 (3434.69)	10286.74 (4016.76)	F=37.516/p<.001*[2,3]
**Right Accumbens**	577.06 (100.18)	534.75 (120.49)	485.29 (119.62)	F=8.402/p<.001*[1,2]
**Right Amygdala**	1637.39 (237.647)	1610.12 (290.03)	1516.35 (226.07)	F=3.497/p=.0325*[2]
**Right Caudate**	3395.69 (464.35)	3469.09 (469.16)	3153.16 (570.51)	F=3.520/p=.0181*[2,3]
**Right Hippocampus**	4128.78 (514.57)	4100.46 (650.54)	3749.94 (708.31)	F=5.081/p=.0093*[2,3]
**Right Pallidum**	1445.26 (183.75)	1583.4 (218.16)	1301.19 (272.39)	F=16.732/p<.001*[2,3]
**Right Putamen**	4725.56 (536.36)	4597.25 (682.25)	3883.06 (1030.05)	F=16.800/p<.001*[2,3]
**Right Thalamus**	6539.22 (649.56)	6815.18 (885.91)	6306.74 (727.66)	F=3.767/p=0.0292*[3]
**Right Ventral DC**	3742.09 (405.7)	3949.57 (534.02)	3493.42 (495.49)	F=8.911/p<.001*[2,3]
**Right Cerebellum**	15846.09 (2840.33)	16190.62 (3031.55)	9200.06 (3487.57)	F=60.91/p<.001*[2,3]

HC: healthy controls; PD: Parkinson's disease patient group; MSA: multiple system atrophy patient group; * refers to FDR corrected results; Post-hoc differences between HC and PD[1]; HC and MSA[2]; PD and MSA[3]; Stats refers to F-test (F).
